# Supplementary material for: Tandem riboswitches form a natural Boolean logic gate to control purine metabolism in bacteria
Source: eLife. 2018 Mar 5;7:e33908. doi: 10.7554/eLife.33908 (PMC5912903; doi:10.7554/eLife.33908)
Supplement: Supplementary file 1. [file elife-33908-supp1.docx]

**Supplemental File 1**

**Tandem riboswitches form a natural Boolean logic gate to control purine metabolism in bacteria**

**Madeline E. Sherlock,^1^ Narasimhan Sudarsan,^2^ Shira Stav,^3^ Ronald R. Breaker^1-3^***

^1^Department of Molecular Biophysics and Biochemistry, ^2^Howard Hughes Medical Institute, ^3^Department of Molecular, Cellular and Developmental Biology, Yale University, P.O. Box 208103, New Haven, CT 06520-8103, USA.

*For correspondence: [ronald.breaker@yale.edu](mailto:ronald.breaker@yale.edu)

Phone: 203 432-9389

**Supplemental Table 1.**

Sequences of synthetic DNAs used in this study.

| Name | Sequence | Annotation |
| --- | --- | --- |
| MES18 | TAATACGACTCACTATAGGTTCACTCATATATGTTTGAGAATATGGCTCAG | Forward PCR primer for transcription of the WT *B. megaterium* 208 *codA* RNA from genomic DNA |
| MES216 | CAATGAGCAAAAACCTACCCGCC | Reverse PCR primer for transcription of the WT *B. megaterium* 208 *codA* RNA from genomic DNA |
| MES131 | TAATACGACTCACTATAGGAAAGTGTATCTAGGGATCCGGAATCAATTCAAGGACCGAGCGGTACAGGTACAACATGTACTACACC | Forward template for transcription of the WT *F. ignava* 106 *purC* RNA |
| MES132 | GGTACGCGAAACCTATCCTCCTGGGCTTTTCTCCCTCTGGTGTAGTACATGTTGTACC | Reverse template for transcription of the WT *F. ignava* 106 *purC* RNA |
| MES48 | GCGCACATGAGAATTCCAGCGACGCTGTTGATCCTTTTAAATAAGTCTGATAAAATGTGAACTAATTGAGCGATCCTTTTTCAAAACTTGAGGGAAGTGCAGCTTAGGGTTCCGCGTGCAACGGCCTGGACCGAGCGCTGCAAAAAGGGGTCAACCGATGATGTTTGCCCTTTTTACACCGGAGGGAGAAAAGCCCAGGCGGATAGGTTCCTTGCATTGGCAAGGCGGGAATCTATCTGCCCGGGCTTTGCTTTTTGCCAAAGGATCCCCAGCTGCGC | IDT G-block containing the wild-type *H. modesticaldum purB* riboswitch and controlled by the *B. subtilis lysC* promoter, for cloning into pDG1661 as well as serving as a template for the dsDNA template for *in vitro* transcription termination |
| MES49 | GCGCACATGAGAATTCCAGCGACGCTGTTGATCCTTTTAAATAAGTCTGATAAAATGTGAACTAATTGAGCGATCCTTTTTCAAAACTTGAGGGAAGTGCAGCTTAGGGTTCCGCGTGCAACGGCCTGGACCGAGCGCTGCAAAAAGGGGTCAACCGATGATGTTTGCCCTTTTTACACCGGAGGGAGAAAAAAACAGGCGGATAGGTTCCTTGCATTGGCAAGGCGGGAATCTAAATAAACGGGCTTTGCTTTTTGCCAAAGGATCCCCAGCTGCGC | IDT G-block containing the terminator mutation (M1) *H. modesticaldum purB* riboswitch and controlled by the *B. subtilis lysC* promoter, for cloning into pDG1661 as well as serving as a template for the dsDNA template for *in vitro* transcription termination |
| MES50 | GCGCACATGAGAATTCCAGCGACGCTGTTGATCCTTTTAAATAAGTCTGATAAAATGTGAACTAATTGAGCGATCCTTTTTCAAAACTTGAGGGAAGTGCAGCTTAGGGTTCCGCGTGCAACGGCCTGGACCGACGGCTGCAAAAAGGGGTCAACCGATGATGTTTGCCCTTTTTACACCGGAGGGAGAAAAGCCCAGGCGGATAGGTTCCTTGCATTGGCAAGGCGGGAATCTATCTGCCCGGGCTTTGCTTTTTGCCAAAGGATCCCCAGCTGCGC | IDT G-block containing the mutation of a conserved nucleotide (M2) *H. modesticaldum purB* riboswitch and controlled by the *B. subtilis lysC* promoter, for cloning into pDG1661 as well as serving as a template for the dsDNA template for *in vitro* transcription termination. |
| MES51 | ACATGAGAATTCCAGCGACGC | Forward primer for amplification of the *H. modesticaldum purB* reporter construct |
| MES52 | AGCTGGGGATCCTTTGGC | Reverse primer for amplification of the *H. modesticaldum purB* reporter construct |
| MES202 | CCAGCGACGCTGTTGATCCTTTTAAATAAGTCTGATAAAATGTGAACTAATTGAGAGATAGTTTTTGAAAAATTGAGGGAAGTGCAGCTTAGGG | Forward primer for the construction of all single-round transcription termination templates from *H. modesticaldum* |
| MES203 | GCGCACATGAGAATTCCAGCGACGCTGCGCAGCTGGGGATCCTTTGGC | Reverse primer for the construction of all single-round transcription termination templates from *H. modesticaldum* |
| MES103 | TACGACGAATTCCAAAAATAATGTTGATCCTTTTAAATAAGTCTGATAAAATGTGAACTAAGAAAAAGTTAAATACGGTT | Forward primer for the construction of *B. megaterium codA* reporter construct as well as single-round transcription termination templates |
| MES104 | GCTCAGGATCCAAATAGACACATAAGAAAA | Reverse primer for the construction of *B. megaterium codA* reporter construct as well as single-round transcription termination templates |
| MES42 | GCTAAATACGGTTCACTCATAATTGTTTGAGAATATGGCTCAG | Forward primer for construction of the *B. megaterium codA* reporter as well as single-round transcription termination template containing M3 via QuikChange mutagenesis |
| MES43 | CTGAGCCATATTCTCAAACAATTATGAGTGAACCGTATTTAGC | Reverse primer for construction of the *B. megaterium codA* reporter as well as single-round transcription termination template containing M3 via QuikChange mutagenesis |
| MES44 | CTACGTTAGTAGAAAGTTTGGTCCAACGGATTCACATATTCATTTTTGAAAAG | Forward primer for construction of the *B. megaterium codA* reporter as well as single-round transcription termination template containing M4 (and M5 using M3 as a the source) via QuikChange mutagenesis |
| MES45 | CTTTTCAAAAATGAATATGTGAATCCGTTGGACCAAACTTTCTACTAACGTAG | Reverse primer for construction of the *B. megaterium codA* reporter as well as single-round transcription termination template containing M4 (and M5 using M3 as a the source) via QuikChange mutagenesis |
